# The in vivo efficacy of neuraminidase inhibitors cannot be determined from the decay rates of influenza viral titers observed in treated patients

**DOI:** 10.1038/srep40210

**Published:** 2017-01-09

**Authors:** John Palmer, Hana M. Dobrovolny, Catherine A. A. Beauchemin

**Affiliations:** 1 Department of Physics, Ryerson University, Toronto, ON, Canada; 2 Department of Physics & Astronomy, Texas Christian University, Fort Worth, TX, USA; 3 Interdisciplinary Theoretical Science (iTHES) Research Group at RIKEN, Wako, Japan

## Abstract

Antiviral therapy is a first line of defence against new influenza strains. Current pandemic preparations involve stock- piling oseltamivir, an oral neuraminidase inhibitor (NAI), so rapidly determining the effectiveness of NAIs against new viral strains is vital for deciding how to use the stockpile. Previous studies have shown that it is possible to extract the drug efficacy of antivirals from the viral decay rate of chronic infections. In the present work, we use a nonlinear mathematical model representing the course of an influenza infection to explore the possibility of extracting NAI drug efficacy using only the observed viral titer decay rates seen in patients. We first show that the effect of a time-varying antiviral concentration can be accurately approximated by a constant efficacy. We derive a relationship relating the true treatment dose and time elapsed between doses to the constant drug dose required to approximate the time- varying dose. Unfortunately, even with the simplification of a constant drug efficacy, we show that the viral decay rate depends not just on drug efficacy, but also on several viral infection parameters, such as infection and production rate, so that it is not possible to extract drug efficacy from viral decay rate alone.

Influenza is a viral infection of the upper respiratory tract that, even in it’s mild seasonal form, causes serious illness and death worldwide^1^. The rapid mutation rate of influenza virus^2^ along with occasional re-assortment events^3^ results in the emergence of new antigenic variants of influenza. The most popular antivirals currently used against influenza are neuraminidase inhibitors (NAIs). NAIs block the action of neuraminidase, an enzyme found on the surface of the influenza virus which is responsible for viral release from an infected cell^4^,^5^ causing virus to remain bound to the cell surface^6^. NAIs, particularly oseltamivir, are the antiviral of choice for pandemic stockpiles^7^ because resistance to NAIs remains low in circulating strains^8^,^9^ and they are effective against pH1N1^10^, as well as avian influenza (H5N1)^11^,^12^. Given our reliance on NAIs and the rapid mutation of influenza into new strains, there is a need to develop methods to rapidly quantify the efficacy of NAIs against new strains of influenza.

There are a variety of techniques used to measure the susceptibilty of a viral strain to NAIs *in vitro*. Perhaps the most direct measure of susceptibility is through a neuraminidase inhibition assay which measures the drug’s effectiveness at inhibiting the neuraminidase activity of a particular viral strain^13^,^14^,^15^,^16^. The inhibition assay, unfortunately, is not equivalent to measuring the drug’s effectiveness at inhibiting viral release during an infection. During an infection virus can be released from the cell without the presence of neuraminidase^17^,^18^,^19^, so we might expect the efficacy of NAIs at blocking an infection to be lower than the efficacy measured in an inhibition assay. To account for this, the efficacy of NAIs is sometimes measured through a reduction in viral yield^20^, cytopathic effect^21^,^22^, or plaque formation^20^,^23^. Knowledge of the within host antiviral efficacy will help in estimating the effect of NAI treatment at the population level, through the use of multiscale models^24^,^25^,^26^, allowing public health officials to manage their stockpiles more effectively.

Influenza infections in humans add further complications. Variability in the metabolism and pharmacokinetics (PK) of drugs between individuals^27^ means that a given dose will have different efficacies in different patients. Thus there is a need to develop methods for determining the effectiveness of NAIs within a human host. Unfortunately, influenza infections are acute and peak quickly around 2 days post-infection. Therefore, characterizing the kinetics of viral titer growth prior to peak is difficult and sometimes impossible as it requires frequent samples within the first 48 of infection, often before patients actually experience symptoms. It is somewhat easier to collect information about the viral titer decay phase which occurs either after the viral titer has peaked or after highly effective treatment sufficient to abrogate the infection is applied. Indeed, several studies have relied on viral measurements starting near or after the viral titer peak to assess the efficacy of NAIs in patients^28^,^29^,^30^,^31^,^32^.

Chronic infections such as those caused by HIV or hepatitis B and C are amenable to extraction of antiviral efficacy from the chronic and relatively constant viral concentration and its ensuing decay following treatment^33^,^34^,^35^. This is because the chronic phase corresponds to a steady state in the viral kinetics (VK) model and the viral titer decay caused by the disruption of that steady state due to antiviral therapy reduces to a function which depends only on the antiviral efficacy and viral clearance rate. Unfortunately, this does not hold in the acute phase of infection because in that phase, infection decay due to antiviral efficacy is dampened by the continued infection growth which characterizes this phase. Yet, perhaps some assumptions could be made here too which would allow us to extract this information from the viral titer decays or perhaps allow us to eliminate some VK parameters from consideration.

Further compounding the problem is the periodic nature of drug treatment. Oseltamivir, the most commonly used NAI, is taken as a pill twice a day which causes fluctuations in the concentration of oseltamivir in the body^27^,^36^. Many VK models simply ignore these fluctuations and assume a constant drug concentration when drug is applied^26^,^37^. The short duration of a seasonal influenza infection means that the fluctuations in drug concentration are on a similar time scale as the infection itself, so it is not clear if the assumption of a constant drug concentration is valid.

In this paper, we use a mathematical model of the within host dynamics of influenza to examine the possibility of extracting drug efficacy from the viral titer decay rate. We first show that viral titers produced with a time-dependent drug concentration can be reproduced with the assumption of a constant drug concentration and that the constant drug assumption results in viral titers with the same viral decay rate as the viral titers produced with a time-dependent drug concentration. We then assess whether constant drug efficacy can be extracted from viral titer decay rate by performing a sensitivity analysis. We find, unfortunately, that the viral decay rate depends on many of the model parameters and we cannot extract drug efficacy without additional data to determine other infection parameters.

## Results

### Fitting treated viral titers

Determining a time-dependent treatment efficacy is difficult particularly given the sparsity of experimental viral titer data. To simplify the problem, we would like to be able to assume that drug efficacy is constant over the course of the infection, but because influenza infections are of short duration (typically 2–4 symptomatic days), oscillations in drug concentration are approximately on the same time scale as the infection. This is not the case for HIV and HCV where this type of analysis has proved useful^33^,^34^,^35^ — these infections last months or even years, so the small oscillations in drug concentration over a few hours are not significant and assuming a constant drug treatment efficacy is reasonable. This might not be the case for a rapidly changing infection such as influenza, so we must first determine whether we can reproduce the viral titer produced by a time-dependent drug treatment with the assumption of a constant drug treatment.

**Figure 1:**
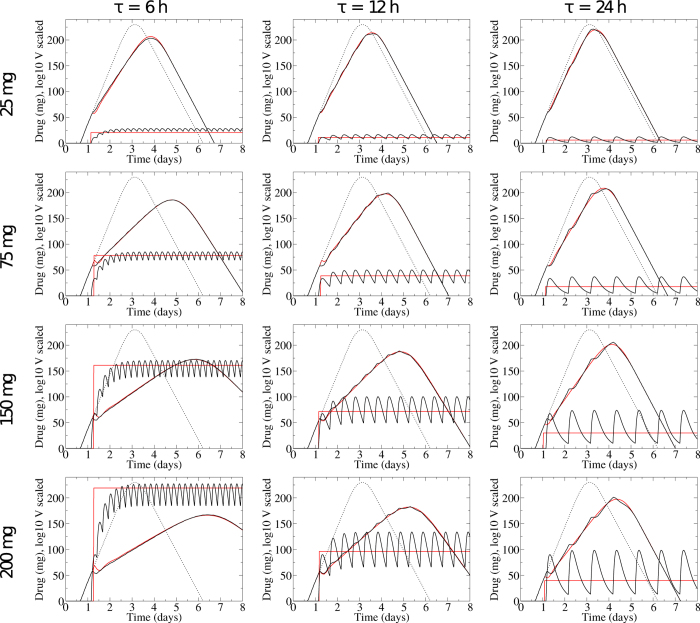
Comparing the effect of a constant vs time-varying antiviral concentration. Viral titers and drug concentrations for infections treated with a time-varying drug concentration, *D*(*t*) from Eq. 4 (black lines), and the best fit assuming a constant drug concentration (red lines). The dashed line shows the untreated infection.

We generate viral titers using time-varying PK/PD models, Eq. (4), with treatment initiated at 28 h. We chose 28 h post-infection, the typical treatment administration time used in human drug trials^38^,^39^, as it is thought to be a few hours after the onset of symptoms and represents a typical time when patients might seek treatment. We then fit the viral titers assuming a constant value for the drug. We leave the constant drug value, *D*_cst_, and the time of administration of the constant drug, *t*_on_, as free parameters. We explore a range of treatment regimens by varying the period of dosage, *τ*, from 0 (theoretical continuous infusion) to 48 h (every two days), and varying the true intake dose, *D*_admin_, from 0 to 200 mg. The typical treatment regimen for oseltamivir is 75 mg twice a day (*τ* = 12 h). Figure 1 shows the viral titers generated with a time-dependent drug concentration and our fits using the assumption of a constant drug concentration.

The viral titer curves obtained by assuming a constant antiviral concentration provide a good approximation of those obtained with the full PK/PD model. There is some discrepancy at high drug doses and large dosage periods (large *D*_admin_ and *τ*) where the oscillations in drug concentration create oscillations in the viral titer. The assumption of a constant drug cannot reproduce these oscillations, so the fits in this range are not as good. Even during these oscillations, however, our fits are within the typical error of viral titer measurements^40^. It is also important to note that viral titers in human trials are typically sampled daily or bi-daily and so these oscillations likely would not be noticed in clinical trial data. There is also some discrepancy near the application time of the drug. In order to match the effect of the drug over the remainder of the viral titer curve, the constant drug needs to be applied at a slightly different time than the time-dependent drug. This leads to a small, experimentally indiscernible difference in the viral titer near the administration time.

**Figure 2:**
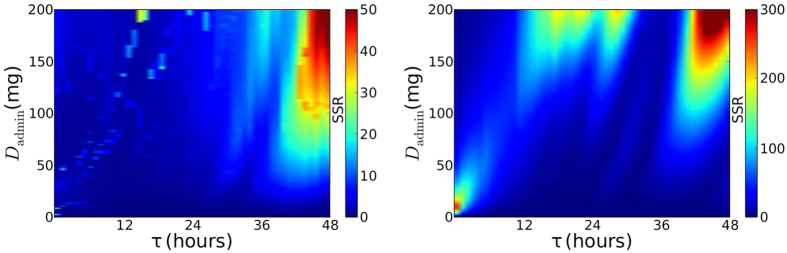
Evaluating the accuracy of assuming a constant drug concentration. The goodness-of-fit (SSR) between viral titers resulting from PK modelling of the time-varying drug concentration and a fit of these titers using a constant drug concentration, *D*_cst_, administered at fixed time, *t*_on_, when (Left) *D*_cst_ and *t*_on_ are fitted; or (Right) *t*_on_ = *t*_admin_ and *D*_cst_ is computed using Eq. (1).

Figure 2 illustrates the goodness of our fits by examining the sum of square residuals (SSR) over the full range of dose concentrations and periods (*D*_admin_ and *τ*). The absolute value for SSR is dependent on the number of data points used in the fit (which was kept constant for all fits) and so the value itself is meaningless, but we can use it as relative measure to determine where we have achieved better fits. Again, the region of high doses and large periods (large *D*_admin_ and *τ*) has larger SSR as oscillations in drug concentration manifest themselves in the viral titer and our constant drug assumption begins to break down. This region does not include the two most common treatment regimens for oseltamivir: 75 mg dose once daily (*τ* = 24 h) for prophylactic therapy or 75 mg twice daily (*τ* = 12 h) for treatment^41^.

### Mapping pharmacokinetic parameters to constant parameters

**Figure 3:**
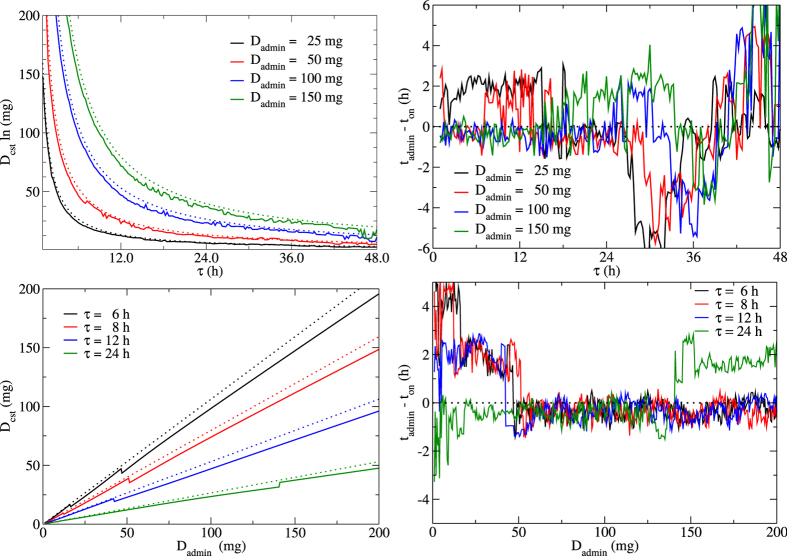
Relationship between parameters of the PK model and those of the constant drug concentration approximation. Relationships between *D*_cst_ and *τ* (upper left), *t*_on_ and *τ* (upper right), *D*_cst_ and *D*_admin_ (bottom left), and *t*_on_ and *D*_admin_ (bottom right). Solid lines indicate actual values determined from fitting; dotted lines indicate our approximations using the constant drug concentration predicted by Eq. 1 and a constant value for *t*_on_.

We determined that assuming a constant drug concentration can provide a good approximation to the full PK model. We must now identify relationships to map the PK parameters (*D*_admin_ and *τ*) to the constant drug parameters (*D*_cst_ and *t*_on_). Figure 3 shows the relationships between the fitted constant parameters (*D*_cst_ and *t*_on_) and the PK parameters (*τ* and *D*_admin_). While there are slight deviations, the constant drug concentration, *D*_cst_, appears to be approximately inversely proportional to the dosage period, *τ*, and linearly proportional to the real dose, *D*_admin_. An expression relating *D*_cst_ to the PK parameters can be derived by using an approximation of the full PK equation. A constant drug can be considered equivalent to a dosage period of *τ* = 0, i.e. no time elapsed between doses as though the drug was continuously applied. Using a Taylor expansion of Eq. (4) about *τ* = 0, we find an approximate expression for *D*_cst_,


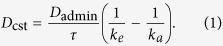


This equation confirms the relationships seen in Fig. 3 (left column) for *D*_cst_, where the dotted lines give the values of *D*_cst_ predicted by Eq. 1. The approximation works best when *τ* and *D*_admin_ are small but still provides a good estimate (within ~10 mg) of *D*_cst_ at high values of both *τ* and *D*_admin_.

In contrast, Fig. 3 (right column) reveals no clear relationship emerging between *t*_on_ and either *D*_admin_ or *τ*, showing small (typically less than 2 h), stochastic variations of *t*_on_ about the true drug administration time, *t*_admin_. The level of stochasticity in the fitted values of *t*_on_ are likely an indication that the infection kinetics are not particularly sensitive to *t*_on_, with several nearby values providing equally good fits, leaving the fitting algorithm to settle on somewhat random neighbouring values. We opted for the simplest assumption, *t*_on_ = *t*_admin_, since *t*_on_ appears to fluctuate about this value as *D*_admin_ and *τ* are varied.

**Figure 4:**
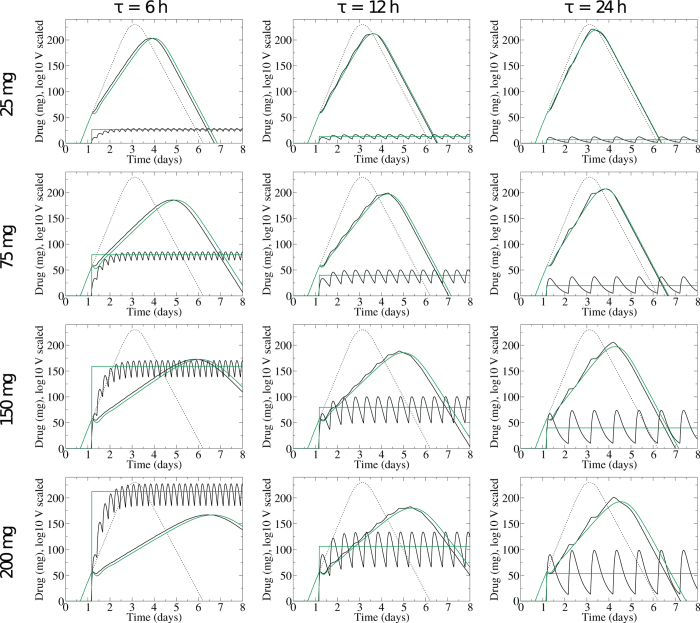
Viral titers and drug concentrations for infections treated with time-varying drug *D*(*t*) from Eq. 4 (black lines), and the viral titers and drug concentrations for infections treated with a constant drug concentration approximation from Eq. 1 and *t*_on_ = *t*_admin_. The dashed line shows the untreated infection.

We now have approximations that allow us to map *D*_admin_ and *τ* to *D*_cst_ and *t*_on_. Figure 4 shows the viral titers and drug concentrations generated by assuming a constant drug concentration determined by Eq. 1 administered at *t*_on_ = *t*_admin_ (green line). The constant drug approximation produces viral titers very close to the viral titers generated with the full PK drug model (black lines). The approximations work well when *τ* and *D*_admin_ are small although there is a slight shift in the time at which the viral titer peaks due to the assumption of a constant *t*_on_ for all *D*_admin_ and *τ*.

We calculated the SSR for the viral titers generated with our approximations (Fig. 2, right). For large *τ*, we still have the problem of oscillations in the PK viral titer that cannot be replicated by a constant drug concentration. The approximation has an additional problem when *D*_admin_ is large and the drug concentration takes a long time to reach steady state. In this case, the assumption that *t*_on_ = *t*_admin_ fails as *D*_cst_ should be applied after *D*_admin_ to account for this prolonged ramp up time. These discrepancies, however, would not be experimentally distinguishable from the PK viral titers due to the inherent error of viral measurement^40^.

**Figure 5:**
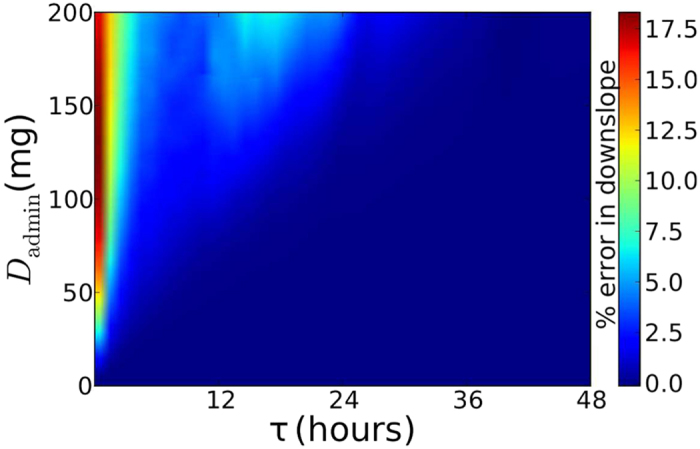
Percent error in decay rate when using a constant drug concentration determined by Eq. (1) and applied at a fixed *t*_on_ = *t*_admin_ to approximate viral titers produced with a time-dependent drug concentration.

While the approximations do not always correctly reproduce every point on the time course, they do appear to accurately reproduce the general shape of the infection. In particular, we are interested in accurately reproducing the slope of the decay phase. Figure 5 shows the percent error in the decay rate. We see that the error is very small over a wide range of doses and dose timings, although there is a small region of more significant error for *τ* < 6 h. In this region, the constant drug assumption leads to a downslope estimate that is smaller than the actual slope so that analysis performed with the constant drug assumption would underestimate the actual efficacy of the drug. It is also unlikely that patients would take pills more often than four times per day, so available clinical trial data will likely be in the region of dose and dose timing where a constant drug assumption leads to a very good estimate of the actual viral decay rate.

### Determining drug efficacy from viral decay

We have now shown that we can reproduce viral titers produced with time-dependent drug treatment under the assumption of a constant drug concentration. More importantly, we have shown that the assumption of a constant drug concentration accurately estimates the slope of the decay phase of viral titer over a range of doses and dose timings. Thus, we can assume a constant drug concentration when attempting to determine the drug efficacy of NAIs. We can now investigate the effect of treatment with oseltamivir on viral titer decay. In our viral kinetics model, we incorporate the effect of treatment with oseltamivir as reducing the production rate of virus by infectious cells such that *p* → (1 − *ε*)*p* with *ε* ∈ [0,1] representing the antiviral efficacy. In the model, *ε* is initially set to zero and is instantaneously set to the desired efficacy at the time of treatment initiation and maintained constant from that time on.

When assessing data from clinical trials, many factors and parameters are unknown. For example, an infection with a given influenza strain within a given patient will be characterized by a set of viral kinetic parameters (e.g., virus production and clearance rate, virus infectivity) which will differ across influenza strains and from patient-to-patient^42^,^43^. Additionally, in the case of recruitment studies, the time at which patients became infected is not known and cannot be determined accurately. It is important, therefore, to evaluate to what extent these unknowns can affect the observed viral titer decay rates from which we hope to determine the efficacy of the antiviral treatment received.

If we can assume that viral load is proportional to infectious cells (*V* = *kI*), then the equation for virus can be solved directly to yield


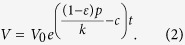


**Figure 6:**
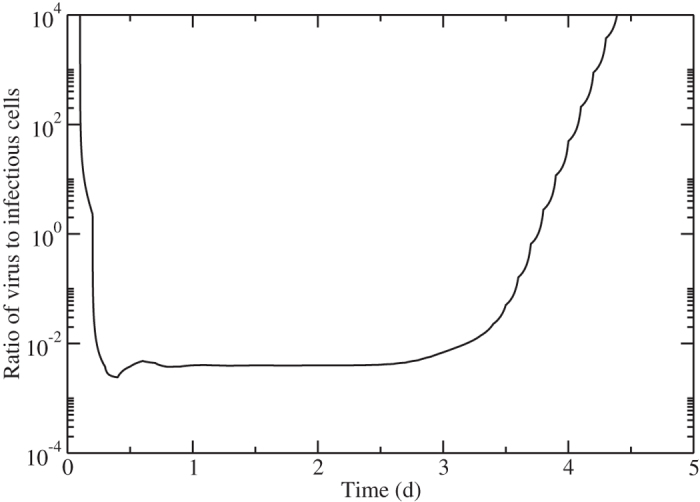
Ratio of viral titer to infectious cells. The ratio of viral titer to infectious cells over the course of an influenza infection.

While *k* remains unknown and likely depends on other viral kinetic parameters, *c* could potentially be estimated from mock infection experiments^43^,^44^, so at least a relative efficacy could be determined from the slope of viral decay. This means we need to determine when the assumption of viral load and infectious cell proportionality holds. While virus is proportional to the number of infectious cells for some parts of the infection, this is not a good approximation for other parts of the cycle, as shown in Fig. 6. In the early stages of infection, there are not yet any infectious cells, so the ratio *V*/*I* is infinite. *V*/*I* decreases rapidly as the number of infectious cells increases, but it doesn’t reach a constant until about 12 hours into the infection. This constant phase lasts until about 3 days post-infection at which point the number of infectious cells starts to decrease again (infectious cells die off faster than virus is cleared), causing *V*/*I* to rise again. In the case of influenza A virus infections *in vitro*, it is known that the decay rate of virus is not proportional to that of infectious or infected cells. That is because the infectious cell lifespan is normally rather than exponentially distributed whereas the lifespan of infectious virus is exponentially distributed^45^. Note that the duration of the roughly 2.5 day period when *V*/*I* is constant will depend on the values of *β, p, c, τ*_*E*_, and *τ*_*I*_, so that in practice, we cannot be entirely sure of the time period when this analysis is applicable. In the case of challenge studies, when time of infection is well-known, it might then be possible to extract efficacy from viral decay, if we are certain that drug treatment is initiated during the phase when *V*/*I* is constant. Note that we cannot use this simplifying assumption for treatment initiated early in the infection when *V*/*I* varies rapidly.

For patients whose time of infection is not known, however, the situation is not so clear-cut. Even supposing that we are certain of when the period of constant *V*/*I* starts and ends, we cannot tell whether we are applying the drug during this time period. Patients typically start to experience symptoms about 1–2 days post-infection^46^,^47^. By the time they get to a doctor and fill a prescription, we can expect them to actually start treatment about 2–3 days post-infection, possibly during the time when *V*/*I* is rising again.

**Figure 7:**
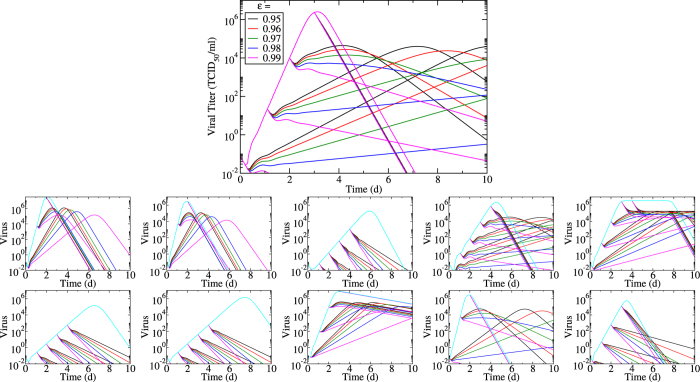
Effect of infection parameters on viral titer decay. Oseltamivir treatment is applied at various times post infection and at different efficacies. Top graph shows the effect of different treatments on the base viral infection parameters. The effect of varying the base infection parameters is also explored by either decreasing (bottom row) or increasing (upper row) viral production rate (*p*) (first column), infection rate (*β*) (second column), viral clearance rate (*c*) (third column), eclipse duration (*τ*_*E*_) (fourth column), infectious lifespan (*τ*_*I*_) (last column) by 10-fold (except *τ*_*E*_, see text) compared to the base parameters.

To investigate how limiting this assumption might be, we examined the viral titer time courses obtained under a variety of antiviral efficacies applied at various times post infection (Fig. 7). We also explore the effect of varying viral infection parameters by varying each parameter by a factor of 10 above and below (with the exception of *τ*_*E*_ which is varied by a factor of two above its base value to prevent unrealistic viral titers) its base value. Note that we have performed the same analysis using a simpler model of influenza infection and found similar results (supplementary material).

We see that viral titer decay is steepest when treatment is applied near viral titer peak. This is because prior to viral peak, viral titer decay due to the suppression of the infection by the antiviral is partly countered by a weakened but persistent infection progression. In contrast, after viral titer peak, viral titer decay occurs irrespective of antiviral treatment. In that phase, decay due to treatment is more pronounced than in the period prior to viral titer peak because at that point all susceptible cells have been consumed and treatment only suppresses viral production in already infected cells which are continuously being lost. In some cases, viral infection parameters are such that even in the presence of treatment, viral titer growth is only subdued and does not immediately result in viral titer decay, but instead results in viral titer growth for a time. Figures showing the effect of antiviral treatment on infectious and eclipse cells are included as supplementary material.

**Figure 8:**
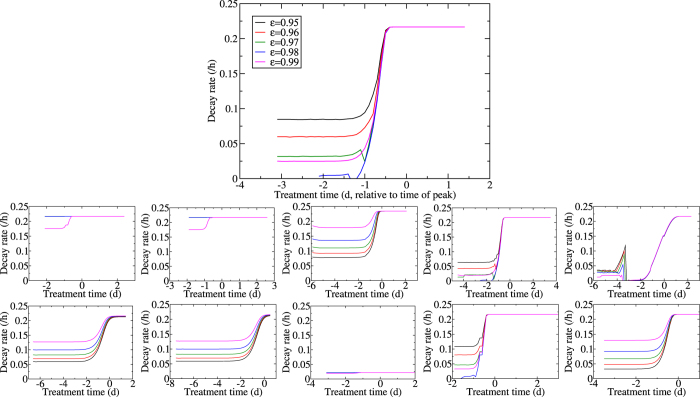
Effect of treatment delay on viral titer decay. Oseltamivir treatment is applied at various times post infection and at different efficacies. The effect of varying the base infection parameters is also explored by either decreasing (bottom row) or increasing (top row) viral production rate (*p*) (first column), infection rate (*β*) (second column), viral clearance rate (*c*) (third column), eclipse duration (*τ*_*E*_) (fourth column), infectious lifespan (*τ*_*I*_) (last column) by 10-fold (except *τ*_*E*_, see text) compared to the base parameters.

Since time of viral titer peak seems to play an important role in viral titer decay rates, we also explored how the latter varies as a function of the treatment time relative to the time of viral titer peak. In Fig. 8, we show the viral titer decay rate, determined by performing a linear regression of ln(*V*) versus time, for each treatment intervention applied at various time relative to viral titer peak. Viral titer decay is steeper when treatment is applied after viral titer peak than prior to it. There is a stronger dependence of viral titer decay on drug efficacy when treatment is started well before the peak. When treatment is applied near the peak this difference becomes smaller and the rate of viral titer decay becomes mostly independent of drug efficacy. In fact, closer examination of the asymptotic value of the viral decay rate after viral titer peak reveals it is equal to the viral clearance rate used in our model, namely 0.22 h^−1^. The viral decay rate after viral titer peak is due solely to the clearance of the virions and not to neuraminidase inhibitor treatment whose inhibition of viral production has become ineffective since most cells have already died. This means that antiviral efficacy cannot be determined from viral decay rates for treatment initiated near or after the peak of the infection. Unfortunately, even if we are confident that treatment was initiated before the viral titer peak, our investigation reveals that the rate of viral titer decay is dependent on all infection parameters. Thus, a higher viral decay rate in one patient could be attributed to either a higher antiviral efficacy in that patient or a lower value in one or more of the viral growth parameters in that patient possibly due to a more effective immune response, or both; these three possibilities are indistinguishable from inspection of viral titer decay alone.

## Discussion

Motivated by studies of HIV and HCV that used mathematical modelling to extract drug treatment efficacy from viral decay rates^33^,^34^,^35^, we investigated the possibility of extracting drug treatment efficacy for influenza infections. Determining drug efficacy for influenza is a slightly more challenging problem because of the short duration and rapidly changing viral titer of influenza. HIV and HCV are chronic infections and drug treatment is typically applied when the infection is in a stable steady state, making it possible to use simplifying assumptions and perform a mathematical analysis of the system. Influenza, on the other hand, does not reach a steady state making mathematical analysis of drug treatment more complex.

One key assumption used in the HIV and HCV analysis is that drug treatment is constant over time. While this is also a common assumption in modelling of influenza infections^26^,^37^,^48^, it has not been determined whether this is actually a reasonable assumption. The typical treatment for influenza involves taking a pill twice a day. This leads to oscillations in the drug concentration. In long-lasting infections such as HIV and HCV, such oscillations are short compared to the duration of the infection. Influenza, however, typically lasts 7 d, so 12 h oscillations in drug concentration might be significant. Herein, we derive an expression (Eq. 1) for converting the time-varying infection dose into a constant drug concentration. We find that with this expression, the approximation of a constant antiviral concentration is appropriate as long as the oscillations in drug concentration are insufficient to create oscillations in the treated viral titers. In particular, we are able to accurately estimate viral decay rate over a broad range of doses and timings under the constant drug assumption. We do not, however, correctly reproduce the time of viral peak; the viral titer curves produced with our constant drug assumptions are shifted in time relative to the original PK viral titers. Our assumptions work best when the true dose (*D*_admin_) and the time elapsed between successive oral doses (*τ*) are not too large so that the time to antiviral concentration steady state is short. When the true drug concentration consists of a series of sparsely spaced spikes, then, not surprisingly, the assumption of a constant drug concentration does not accurately reproduce viral titer kinetics.

Since we found that assuming a constant drug concentration for modelling of influenza infections is a reasonable assumption, we used the simplification of a constant drug assumption to assess whether it is possible to extract drug efficacy from the viral decay rate alone. A previous study derived an analytical expression for the viral decay rate of untreated influenza infections modelled using exponential transitions between cell states^49^. This study determined that the viral decay rate is determined by the smallest of *c*, 1/*τ*_*I*_, or 1/*τ*_*E*_. Our model is based on more realistic probability distributions for cell transitions^50^ which does not have this same dependency. Our application of drug also complicates the analysis since we are sometimes imposing viral decay when an untreated infection would still be growing. A simplifying assumption of viral load and infectious cell proportionality is possible for influenza, but our analysis showed that this assumption only holds for about 60 h during the course of the infection. Since we often do not know when the infection started, it is difficult to judge whether this assumption is valid for the patient we are assessing. However, the key point of our manuscript is not whether in theory the efficacy could be extractable provided that certain conditions are met. Rather, the key point is whether the efficacy of an antiviral drug can reliably be determined from viral titer decay alone. For example, viral titer decay alone cannot confirm (or deny) that V is proportional to I during said decay and assuming that it is would not be appropriate unless other knowledge/information is available (i.e. not just viral titer decay data alone). While others might have made this assumption in the past and might have claimed to have estimated antiviral efficacy via this assumption, then it is their claim that is too strong. That is, unless they provided additional evidence (i.e. beyond viral decay data alone) that *I* is indeed proportional to V during decay for their particular virus/host system.

Moreover, it is clear that the rate of decay of viral titer in this model is determined not only by drug efficacy, but also by the viral infection parameters and the time at which treatment is initiated. Similar parameter dependencies have been observed in chronic infections^51^ although for chronic infections it is often possible to estimate these parameters with data collected from the chronic phase of the infection. In the case of influenza, we often only have clinical data from the decay phase, so it is difficult to estimate infection parameters. This means that a larger viral decay rate in a patient could be attributed to either or both increased antiviral efficacy and decreased viral titer growth parameters. Thus, not surprisingly, antiviral efficacy cannot be determined from viral titer decay alone and requires knowledge of the parameters driving infection growth in each patient. To establish these parameters, viral titers at the early time of the infection (i.e., during viral titer growth) are required in sufficient number to determine the infection growth rate (i.e., at least 3 viral titer samples prior to viral titer peak to reasonably characterize the viral titer up-slope).

Another key concern in determining antiviral efficacy is distinguishing viral decay due to antiviral treatment from that due to the natural resolution of the infection. We found applying antiviral treatment later results in larger viral titer decay rates. Since influenza is an acute infection, it typically resolves on its own and the viral decay after viral peak occurs even in the absence of antiviral treatment. That natural viral decay rate is likely modulated by the strength of the immune response in clearing both infected cells and free virus. Therefore, viral titer decay in treated patients can be due to either or both natural resolution of the infection and the action of the antiviral. As such, even with a comprehensive, well-sampled, viral titer time course, it might not be possible to resolve antiviral efficacy in a given patient as it would be indistinguishable from natural infection resolution. Studies where decay phase of the viral load are used to examine the efficacy of NAIs^28^,^29^,^30^,^31^,^32^ are tacitly assuming that all their patients can be described with the same infection parameters, particularly that they would have the same natural decay rate in the absence of antivirals. This is not a reasonable assumption, and our study shows that even small variations in underlying dynamical parameters will alter the measured viral decay rate. We believe antiviral efficacy in treating influenza is best determined from massive sets of viral titers and would manifest therein as a statistically significant departure of the viral kinetic parameters of a treated group versus that of an untreated group. Perhaps sub-optimal treatment in some of the antiviral-treated group would help show a progression or shift in the viral kinetics parameter(s) from the placebo-treated to the effectively treated patient groups which could make a more convincing case for the efficacy of the antiviral in affecting that viral kinetic parameter.

## Methods

### Viral kinetic model

We use a modified version^50^ of the basic influenza infection model first presented in ref. 48.


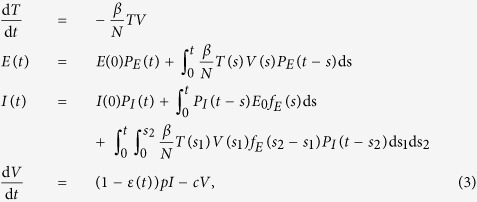


Target cells (*T*) are infected at a rate *β*/*N*, where *N* is the total number of cells, and enter the eclipse phase. Eclipse cells (*E*) are cells that have been infected by virus (*V*) but are not yet releasing any new virions. Infectious cells (*I*) are producing and releasing virus at a rate *p*. Infectious cells eventually stop producing virus (and/or die) and are removed from the system. Virus is removed at clearance rate *c*. Transition from eclipse to infectious is a probabilistic process where *P*_*E*_(*t*) (or *P*_*I*_(*t*)) is the probability that the cell remains in the eclipse (or infectious) phase for a time *t*, and *f*_*E*_(*t*) is the associated probability density function. We model both transitions with normal distributions having standard deviations *σ*_*E*_ or *σ*_*I*_ and average lifespans *τ*_*E*_ and *τ*_*I*_. The use of the normal distribution is more biologically realistic than the exponential distribution tacitly assumed by ordinary differential equations, as the normal distribution imposes a minimum length of time in which the cell must remain in that state and does not allow cells to remain in a particular state for excessively long times^50^.

We model the action of NAIs, which block viral release^4^,^5^, as reducing the production rate *p*. The drug is applied via the parameter *ε*(*t*), which is a number between 0 and 1 and represents the potentially time-varying efficacy of the drug. Viral kinetic parameters were taken from ref. 48 and are presented in Table 1.

### Pharmacokinetic and pharmacodynamic models

Our aim is to determine whether the viral titer resulting from a time-varying drug treatment can be approximated by viral titers produced with the assumption of constant drug treatment. For this, we need a time-varying PK model to simulate time-varying drug treatment regimens. We use the following PK model to simulate the course of a time-varying drug regimen^52^,


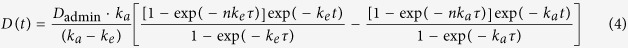


where *D*_admin_ is the dose of the pill administered in units of mg, *τ* is the time between doses, *k*_*a*_ and *k*_*e*_ are the drug absorption and elimination rates, respectively, and *n* is the number of doses administered. Time, *t*, is measured from the *n*^th^ dose. We use the pharmacokinetic (PK) parameters for oseltamivir because it is the most commonly prescribed NAI^53^. The parameters were obtained from studies of oseltamivir PK^54^ and are presented in Table 1. Note that we are not trying to reproduce the full pharmacokinetics of oseltamivir, which as a prodrug would require a two compartment PK model^55^, but are trying to ascertain whether a time-varying drug regimen can reasonably be approximated by an assumption of a constant level of drug.

The drug efficacy used in the viral kinetics model is related to drug dose through the E_max_ pharmacodynamic (PD) model^56^,


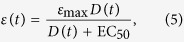


where EC_50_ is the dose at which half the maximal effect is achieved and *ε*_max_ is the maximum effect of the drug. Efficacy is between 0 and 1 and represents the fractional reduction in production from an untreated infection. For example, an efficacy of 0.4 reduces the viral production rate by 40%. In the case of a time-varying drug dose, and thus a time-varying drug efficacy, the effective production rate will also vary in time. Oseltamivir binds to and inhibits the activity of neuraminidase^4^,^5^, as the amount of drug available in the system varies with time, the number of bound, deactivated neuraminidase proteins will also vary, altering the viruses ability to release itself from the cell surface.

The EC_50_ for oseltamivir is dependent on the viral strain and so it can vary widely, from 0.0008–35 μM^57^. We decided to use 0.5 μM as it is approximately in the middle of this wide range. Note that we are using EC_50_ rather than IC_50_ because the viral kinetics model models the effect of the drug on the course of the infections and not inhibition of NA activity by the drug^58^. Drug efficacy is a unitless quantity so drug dose can be represented either as the oral dose or as the blood plasma concentration with no loss in generality as long as EC_50_ is represented in the same units. Since we are modelling *in vivo* human infections which are treated by giving pills with doses in milligrams, we convert the EC_50_ to milligrams based on parameters from ref. 54 for ease of interpretation. There have not, to date, been any studies that have determined *ε*_max_ for oseltamivir. Instead, we use the results of previous modelling studies of zanamivir (a drug in the same class as oseltamivir) treatment of influenza infections^26^,^48^ and set *ε*_max_ = 0.98.

## Additional Information

**How to cite this article**: Palmer, J. *et al*. The in vivo efficacy of neuraminidase inhibitors cannot be determined from the decay rates of influenza viral titers observed in treated patients. *Sci. Rep.* **7**, 40210; doi: 10.1038/srep40210 (2017).

**Publisher's note:** Springer Nature remains neutral with regard to jurisdictional claims in published maps and institutional affiliations.

## Supplementary Material

Supplementary Information

## Figures and Tables

**Table 1:** Parameters and initial conditions taken from ref. 48 unless otherwise indicated.

Parameter	Meaning	Value
Viral kinetic parameters		
*β*	Infection rate	3.2 × 10^−5^ d^−1^ ⋅ [TCID_50_/mL]^−1^
*p*	Production rate	4.6 × 10^−2^TCID_50_⋅d^−1^⋅mL^−1^
*c*	Clearance rate	5.2 d^−1^
*τ*_*E*_	Eclipse phase, mean duration	6 h
*σ*_*E*_	Eclipse phase, standard deviation	1 h^45^
*τ*_*I*_	Infectious lifespan, mean duration	12 h^45^
*σ*_*I*_	Infectious lifespan, standard deviation	1 h^45^
Initial conditions		
*T*_0_	Initial number of target cells	4 × 10^8^cells
*E*_0_	Initial number of eclipse cells	0 cells
*I*_0_	Initial number of infectious cells	0 cells
*V*_0_	Initial amount of virus	7.5 × 10^−2^TCID_50_
Pharmacokinetic/pharmacodynamic parameters		
*k*_*a*_	Drug absorption rate	0.46 h^−1 ^54
*k*_*e*_	Drug elimination rate	0.11 h^−1 ^54
EC_50_	50% effective concentration	7.3 mg [see text]
*ε*_max_	Maximum inhibitory effect	0.98 [see text]

## References

[b1] Centres for Disease Control and Prevention (CDC). Influenza activity – United States and worldwide, 2007–08 season. Morb Mortal Wkly Rep 57, 692–697 (2008). 18583957

[b2] Drake J. W. Rates of spontaneous mutation among RNA viruses. Proc. Natl. Acad. Sci. USA 90, 4171–4175 (1993). 8387212 10.1073/pnas.90.9.4171 PMC46468

[b3] Ghedin E. . Mixed infection and the genesis of influenza virus diversity. J. Virol. 83, 8832–8841 (2009). 19553313 10.1128/JVI.00773-09 PMC2738154

[b4] Abed Y. . Characterization of 2 influenza A(H3N2) clinical isolates with reduced susceptibility to neuraminidase inhibitors due to mutations in the hemagglutinin gene. J. Infect. Dis. 186, 1074–1080 (2002). 12355356 10.1086/344237

[b5] Gubareva L. V., Kaiser L. & Hayden F. G. Influenza virus neuraminidase inhibitors. Lancet 355, 827–835 (2000). 10711940 10.1016/S0140-6736(99)11433-8

[b6] Gubareva L. V. . Characterization of mutants of influenza A virus selected with the neuraminidase inhibitor 4-guanidino-Neu5Ac2en. J. Virol. 70, 1818–1827 (1996). 8627706 10.1128/jvi.70.3.1818-1827.1996 PMC190009

[b7] Schünemann H. J. . WHO rapid advice guidelines for pharmacological management of sporadic human infection with avian influenza A (H5N1) virus. Lancet Infect. Dis. 7, 21–31 (2007). 17182341 10.1016/S1473-3099(06)70684-3 PMC7106493

[b8] Baz M., Abed Y., McDonald J. & Boivin G. Characterization of multidrug-resistant influenza A/H3N2 viruses shed during 1 year by an immunocompromised child. Clin. Infect. Dis. 43, 1555–1561 (2006). 17109288 10.1086/508777

[b9] Weinstock D. M. & Zucotti G. The evolution of influenza resistance and treatment. JAMA 301, 1066–1069 (2009). 19255112 10.1001/jama.2009.324

[b10] Kawai N. . Comparison of the clinical symptoms and the effectiveness of neuraminidase inhibitors for patients with pandemic influenza H1N1 2009 or seasonal H1N1 influenza in the 2007–2008 and 2008–2009 seasons. J. Infect. Chemother. 17, 375–381 (2011). 21120678 10.1007/s10156-010-0179-9

[b11] Rameix-Welti M.-A., van der Werf S. & Naffakh N. Sensitivity of H5N1 influenza viruses to oseltamivir: An update. Future Virol. 3, 157–165 (2008).

[b12] de Jong M. D. . Oseltamivir resistance during treatment of influenza A (H5N1) infection. N. Engl. J. Med. 353, 2667–2672 (2005). 16371632 10.1056/NEJMoa054512

[b13] Wetherall N. T. . Evaluation of neuraminidase enzyme assays using different substrates to measure susceptibility of influenza virus clinical isolates to neuraminidase inhibitors: Report of the neuraminidase inhibitor susceptibility network. Journal of Clinical Microbiology 41, 742–750 (2003). 12574276 10.1128/JCM.41.2.742-750.2003 PMC149673

[b14] Gubareva L. V., Webster R. G. & Hayden F. G. Detection of influenza virus resistance to neuraminidase inhibitors by an enzyme inhibition assay. Antiviral Res. 53, 47–61 (2002). 11684315 10.1016/s0166-3542(01)00192-9

[b15] Nguyen J. T., Sheu T. G., Mishin V. P., Klimov A. I. & Gubareva L. V. Assessment of pandemic and seasonal influenza A (H1N1) virus susceptibility to neuraminidase inhibitors in three enzyme activity inhibition assays. Antimicrob. Agents Chemother. 54, 3671–3677 (2010). 20585136 10.1128/AAC.00581-10 PMC2934949

[b16] Okomo-Adhiambo M., Sheu T. G. & Gubareva L. V. Assays for monitoring susceptibility of influenza viruses to neuraminidase inhibitors. Influenza and Other Resp. Viruses 7, 44–49 (2012). 10.1111/irv.12051 PMC5978623 23279896

[b17] Gubareva L. V. . A release-competent influenza a virus mutant lacking the coding capacity for the neuraminidase active site. J. Gen. Virol. 83, 2683–2692 (2002). 12388803 10.1099/0022-1317-83-11-2683

[b18] Hughes M. T., Matrosovich M., Rodgers M. E., McGregor M. & Kawaoka Y. Influenza A viruses lacking sialidase activity can undergo multiple cycles of replication in cell culture, eggs, or mice. J. Virol. 74, 5206–5212 (2000). 10799596 10.1128/jvi.74.11.5206-5212.2000 PMC110874

[b19] Liu C., Eichelberger M. C., Compans R. W. & Air G. M. Influenza type A virus neuraminidase does not play a role in viral entry, replication, assembly, or budding. J. Virol. 69, 1099–1106 (1995). 7815489 10.1128/jvi.69.2.1099-1106.1995 PMC188682

[b20] Bauer K. . Neuraminidase inhibitor susceptibility of swine influenza A viruses isolated in germany between 1981 and 2008. Med. Microbiol. and Immunol. 201, 61–72 (2012). 21688167 10.1007/s00430-011-0206-1

[b21] Hoopes J. D. . Triple combination antiviral drug (TCAD) composed of amantadine, oseltamivir, and ribavirin impedes the selection of drug-resistant influenza A virus. PLoS ONE 6, e29778 (2011). 22220216 10.1371/journal.pone.0029778 PMC3248427

[b22] Nguyen J. T. . Triple combination of oseltamivir, amantadine, and ribavirin displays synergistic activity against multiple influenza virus strains *in vitro*. Antimicrob. Agents Chemother. 53, 4115–4126 (2009). 19620324 10.1128/AAC.00476-09 PMC2764153

[b23] Samson M. . Characterization of drug-resistant influenza virus A(H1N1) and A(H3N2) variants selected *in vitro* with laninamivir. Antimicrob. Agents Chemother. 58, 5220–5228 (2014). 24957832 10.1128/AAC.03313-14 PMC4135884

[b24] Dang Y.-X., Li X.-Z. & Martcheva M. Competitive exclusion in a multi-strain immuno-epidemiological influenza model with environmental transmission. J. Biol. Dyn. 10, 416–456 (2016). 27608293 10.1080/17513758.2016.1217355

[b25] Pawelek K. A., Salmeron C. & Valle S. D. Connecting within and between-hosts dynamics in the influenza infection-staged epidemiological models with behavior change. J. Coupled Systems Multiscale Dyn. 3, 233–243 (2015). 10.1166/jcsmd.2015.1082 PMC5654582 29075652

[b26] Handel A., Longini Jr, I. M. & Antia R. Neuraminidase inhibitor resistance in influenza: Assessing the danger of its generation and spread. PLoS Comput. Biol. 3, e240 (2007). 18069885 10.1371/journal.pcbi.0030240 PMC2134965

[b27] He G., Massarella J. & Ward P. Clinical pharmacokinetics of the prodrug oseltamivir and its active metabolite Ro 64-0802. Clin. Pharmacokinet. 37, 471–484 (1999). 10628898 10.2165/00003088-199937060-00003

[b28] Zhang Y. . Efficacy of oseltamivir-peramivir combination therapy compared to oseltamivir monotherapy for influenza A (H7N9) infection: a retrospective study. BMC Infect. Dis. 16, 76 (2016). 26864456 10.1186/s12879-016-1383-8 PMC4748590

[b29] Whitley R. . Single dose peramivir for the treatment of acute seasonal influenza: integrated analysis of efficacy and safety from two placebo-controlled trials. Antiviral Therapy 20, 709–719 (2015). 25318121 10.3851/IMP2874

[b30] Sugaya N. . Comparison between virus shedding and fever duration after treating children with pandemic A H1N1/09 and children with A H3N2 with a neuraminidase inhibitor. Antivir. Ther. 20, 49–55 (2015). 24832015 10.3851/IMP2798

[b31] Sato M. . Sequential influenza B viral load and susceptibility in children treated with oseltamivir and zanamivir. Ped. Inf. Dis. J 33, E168–E172 (2014). 10.1097/INF.0000000000000266 24445826

[b32] Takemoto Y. . Clinical effects of oseltamivir, zanamivir, laninamivir and peramivir on seasonal influenza infection in outpatients in japan during the winter of 2012–2013. Chemother. 59, 373–378 (2013). 10.1159/000362436 24821568

[b33] Neumann A. U. . Hepatitis C viral dynamics *in vivo* and the antiviral efficacy of interferon-*α* therapy. Science 282, 103–107 (1998). 9756471 10.1126/science.282.5386.103

[b34] Nowak M. A. . Viral dynamics in hepatitis B virus infection. Proc. Natl. Acad. Sci. USA 93, 4398–4402 (1996). 8633078 10.1073/pnas.93.9.4398 PMC39549

[b35] Perelson A. S., Neumann A. U., Markowitz M., Leonard J. M. & Ho D. D. HIV-1 dynamics *in vivo*: Virion clearance rate, infected cell life-span, and viral generation time. Science 271, 1582–1586 (1996). 8599114 10.1126/science.271.5255.1582

[b36] Massarella J. W. . The pharmacokinetics and tolerability of the oral neuraminidase inhibitor oseltamivir (Ro 64-0796/GS4104) in healthy adult and elderly volunteers. J. Clin. Pharmacol. 40, 836–843 (2000). 10934667 10.1177/00912700022009567

[b37] Dobrovolny H. M., Gieschke R., Davies B. E., Jumbe N. L. & Beauchemin C. A. A. Neuraminidase inhibitors for treatment of human and avian strain influenza: A comparative study. J. Theor. Biol. 269, 234–244 (2011). 20970433 10.1016/j.jtbi.2010.10.017

[b38] Hayden F. G. . Use of the oral neuraminidase inhibitor oseltamivir in experimental human influenza: randomized controlled trials for prevention and treatment. JAMA 282, 1240–1246 (1999). 10517426 10.1001/jama.282.13.1240

[b39] Gubareva L. V., Kaiser L., Matrosovich M. N., Soo-Hoo Y. & Hayden F. G. Selection of influenza virus mutants in experimentally infected volunteers treated with oseltamivir. J. Infect. Dis. 183, 523–531 (2001). 11170976 10.1086/318537

[b40] LaBarre D. D. & Lowy R. J. Improvements in methods for calculating virus titer estimates from TCID_50_ and plaque assays. J. Virol. Methods 96, 107–126 (2001). 11445142 10.1016/s0166-0934(01)00316-0

[b41] Widmer N. . Oseltamivir in seasonal, avian H5N1 and pandemic 2009 A/H1N1 influenza: Pharmacokinetic and pharmacodynamic characteristics. Clin. Pharmacokinet. 49, 741–765 (2010). 20923248 10.2165/11534730-000000000-00000

[b42] Holder B. P. . Assessing the *in vitro* fitness of an oseltamivir-resistant seasonal A/H1N1 influenza strain using a mathematical model. PLoS ONE 6, e14767 (2011). 21455300 10.1371/journal.pone.0014767 PMC3063785

[b43] Pinilla L. T., Holder B. P., Abed Y., Boivin G. & Beauchemin C. A. A. The H275Y neuraminidase mutation of the pandemic A/H1N1 influenza virus lengthens the eclipse phase and reduces viral output of infected cells, potentially compromising fitness in ferrets. J. Virol. 86, 10651–10660 (2012). 22837199 10.1128/JVI.07244-11 PMC3457267

[b44] Beauchemin C. A. A. . Modeling amantadine treatment of influenza A virus *in vitro*. J. Theor. Biol. 254, 439–451 (2008). 18653201 10.1016/j.jtbi.2008.05.031 PMC2663526

[b45] Holder B. P., Liao L. E., Simon P., Boivin G. & Beauchemin C. A. A. Design considerations in building in silico equivalents of common experimental influenza virus assays and the benefits of such an approach. Autoimmunity 44 (2011). 10.3109/08916934.2011.523267 21244331

[b46] Hayden F. G. . Local and systemic cytokine responses during experimental human influenza A virus infection. Relation to symptom formation and host defense. J. Clin. Invest. 101, 643–649 (1998). 9449698 10.1172/JCI1355 PMC508608

[b47] Fritz R. S. . Nasal cytokine and chemokine response in experimental influenza A virus infection: Results of a placebo-controlled trial of intravenous zanamivir treatment. J. Infect. Dis. 180, 586–593 (1999). 10438343 10.1086/314938

[b48] Baccam P., Beauchemin C., Macken C. A., Hayden F. G. & Perelson A. S. Kinetics of influenza A virus infection in humans. J. Virol. 80, 7590–7599 (2006). 16840338 10.1128/JVI.01623-05 PMC1563736

[b49] Smith A. M., Adler F. R. & Perelson A. S. An accurate two-phase approximate solution to an acute viral infection model. J. Math. Biol. 60, 711–726 (2010). 19633852 10.1007/s00285-009-0281-8 PMC2841722

[b50] Holder B. P. & Beauchemin C. A. A. Exploring the effect of implementing different biological delays in constructing kinetic models of influenza infection within a host or cell culture. BMC Public Health 11, S10 (2011). 10.1186/1471-2458-11-S1-S10 PMC3317580 21356129

[b51] Lloyd A. L. The dependence of viral parameter estimates on the assumed viral life cycle: Limitations of studies of viral load data. Proc. Biol. Sci. 268, 847–854 (2001). 11345331 10.1098/rspb.2000.1572 PMC1088679

[b52] Dhillon S. & Kostrzewski A. (eds) Clinical Pharmacokinetics 1st edn. (Pharmaceutical Press, London, 2006).

[b53] Hsieh Y.-H., Chen K.-F., Gaydos C. A., Rothman R. E. & Kelen G. D. Antiviral prescriptions to US ambulatory care visits with a diagnosis of influenza before and after high level of adamantane resistance 2005–06 season. PLoS One 5, e8945 (2010). 20126611 10.1371/journal.pone.0008945 PMC2812486

[b54] Rayner C. R., Chanu P., Gieschke R., Boak L. M. & Jonsson E. N. Population pharmacokinetics of oseltamivir when coadministered with probenecid. J. Clin. Pharmacol. 48, 935–947 (2008). 18524996 10.1177/0091270008320317

[b55] Davies B. E. Pharmacokinetics of oseltamivir: an oral antiviral for the treatment and prophylaxis of influenza in diverse populations. J. Antimicrob. Chemother. 65, 5–10 (2010). 10.1093/jac/dkq015 PMC2835511 20215135

[b56] Holford N. H. G. & Sheiner L. B. Understanding the dose-effect relationship: Clinical application of pharmacokinetic-pharmacodynamic models. Clin. Pharmacokinet. 6, 429–453 (1981). 7032803 10.2165/00003088-198106060-00002

[b57] Roche Pharmaceuticals. Tamiflu (oseltamivir phosphate) capsules and for oral suspension. Roche Laboratories Inc. (2005).

[b58] Beggs N. F. & Dobrovolny H. M. Determining drug efficacy parameters for mathematical models of influenza. J. Biol. Dynamics 9, 332–346 (2015). 10.1080/17513758.2015.1052764 26056712

