# Supplementary material for: The in vivo efficacy of neuraminidase inhibitors cannot be determined from the decay rates of influenza viral titers observed in treated patients
Source: Sci Rep. 2017 Jan 9;7:40210. doi: 10.1038/srep40210 (PMC5220315; doi:10.1038/srep40210)
Supplement: Supplementary Information [file srep40210-s1.pdf]

Supplementary material for "The in vivo efficacy of neuraminidase inhibitors cannot be determined from the decay rates of influenza viral titers observed in treated patients"

John Palmer, Hana M. Dobrovolny, Catherine A.A. Beauchemin

1 Effect of antiviral treatment on eclipse and infectious cells

In the main text, we show the effect of antiviral treatment on viral titer. In this supplement, we include the graphs showing the effect of viral titer treatment on the eclipse and infectious cells (Figs. 1 & 2). There are experimental techniques that allow measurement of infected cells (1–3), but they do not discriminate between cells in the eclipse phase and those in the infectious phase. We see, however, that even if we measure the time course of infected cells, the decay rate alone will not provide sufficient information to extract the efficacy of the antiviral. The slope of the decay phase changes not only with drug efficacy, but also changes as base infection parameters change.

2 Estimating drug efficacy for a TIV model

Results in the main paper were based on simulations of a model with normally-distributed transitions between the eclipse and infectious phases as well as between the infectious and dead phases. It could be that our results are specific to this particular model. In this supplement, we present simulation results for a simpler infection model that includes only target cells, infectious

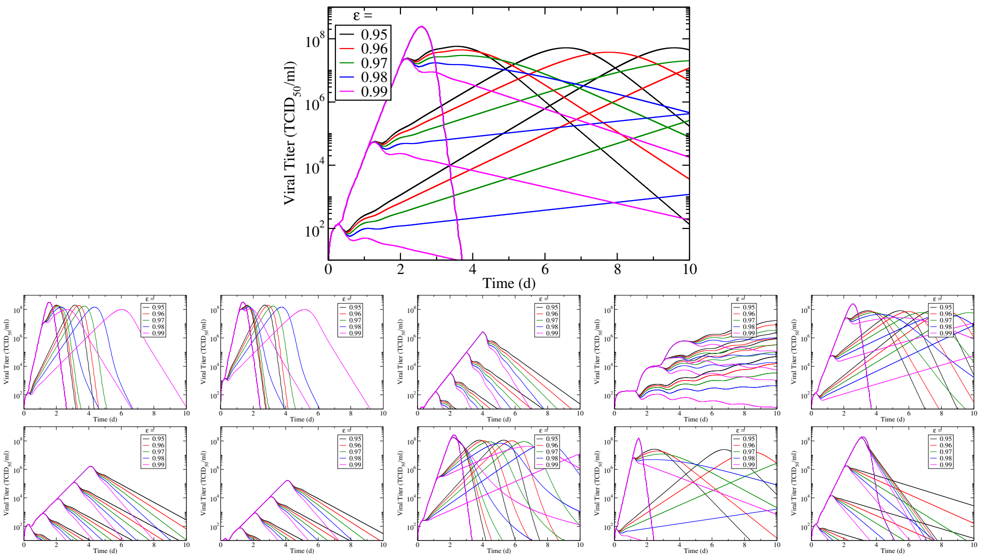

Figure 1: Effect of infection parameters on cells in the eclipse phase. Oseltamivir treatment is applied at various times post infection and at different efficacies. Top graph shows the effect of different treatments on the base viral infection parameters. The effect of varying the base infection parameters is also explored by either decreasing (bottom row) or increasing (upper row) viral production rate (p) (first column), infection rate (β) (second column), viral clearance rate (c) (third column), eclipse duration (fourth column), or infectious lifespan (τ_I) (last column) by 10-fold compared to the base parameters.

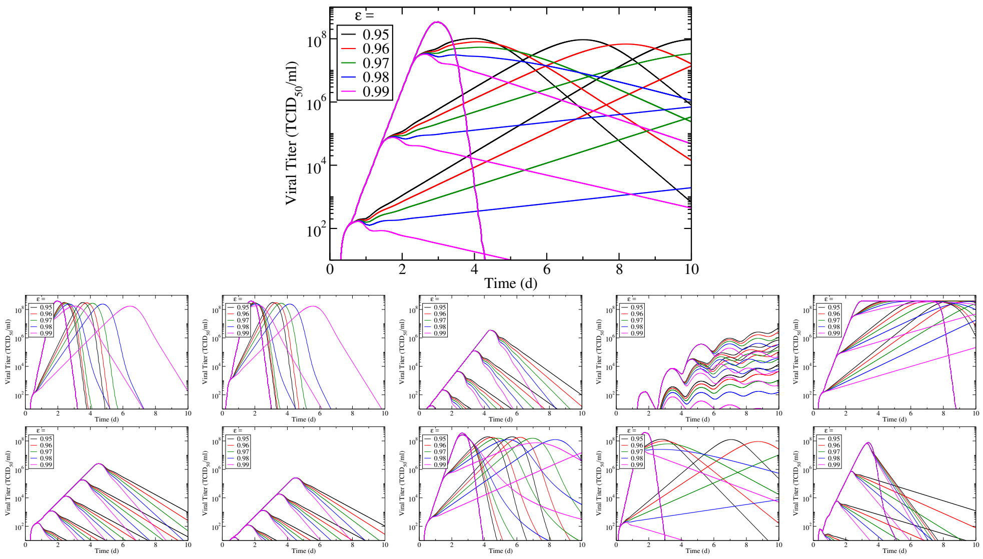

Figure 2: Effect of infection parameters on cells in the infectious phase. Oseltamivir treatment is applied at various times post infection and at different efficacies. Top graph shows the effect of different treatments on the base viral infection parameters. The effect of varying the base infection parameters is also explored by either decreasing (bottom row) or increasing (upper row) viral production rate (p) (first column), infection rate (β) (second column), viral clearance rate (c) (third column), eclipse duration (fourth column), or infectious lifespan (τ_I) (last column) by 10-fold compared to the base parameters.

Table 1: Parameter values for the TIV model taken from Baccam et al. (4).

Parameter	Value
β	2.7×10^{-5} $\mathrm{TCID}_{50}^{-1}\mathrm{d}^{-1}$
p	1.2×10^{-2} $\mathrm{TCID}_{50}\mathrm{d}^{-1}$
δ	4.0 /d
c	3.0 /d
V_0	9.3×10^{-2} TCID_{50}

cells, and virus,

$$\begin{aligned} \frac{\mathrm{d}T}{\mathrm{d}t} &= -\beta TV \\ \frac{\mathrm{d}I}{\mathrm{d}t} &= \beta TV - \delta I \\ \frac{\mathrm{d}V}{\mathrm{d}t} &= pI - cV. \end{aligned} \tag{1}$$

For simulations of this model, we use parameters determined by fits of this model to patient data (Table 1) (4).

We plot the viral titers for treatment applied with different time delays and with different efficacies in Fig. 3. As in the main text, the figure also shows the viral titers when base parameters are varied ten-fold. Fig. 4 shows the decay rates calculated as a function of the treatment delay. We find that the results for the TIV model are similar to the results found in the main text with some dependence of decay rate on drug efficacy as long as treatment is applied before the viral peak, but no dependence on drug efficacy if treatment is applied after the peak. Since we often do not know whether we are applying the drug before or after the peak, we cannot determine drug efficacy from this measurement.

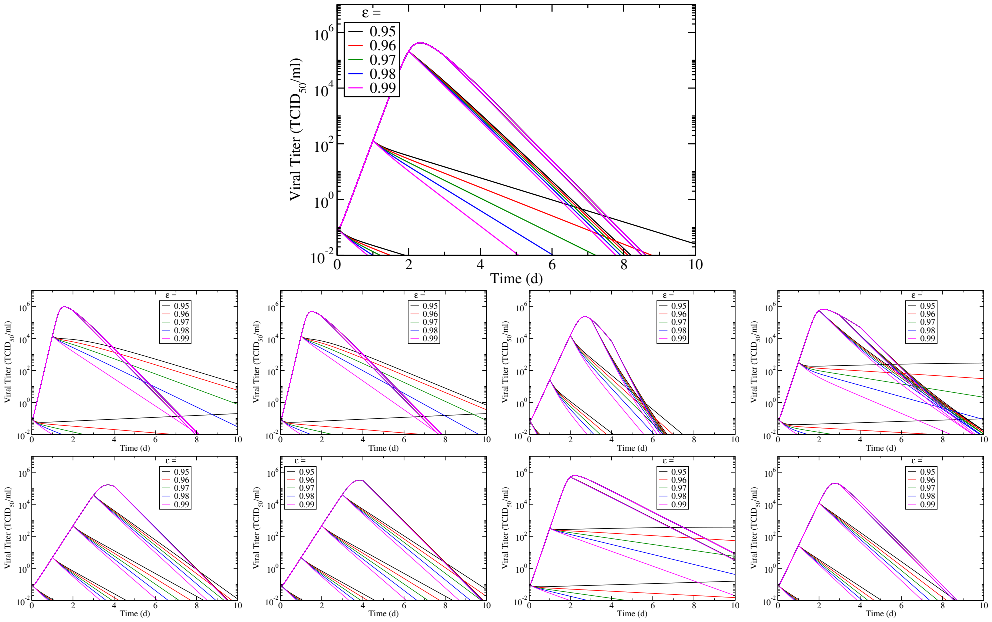

Figure 3: Effect of infection parameters on viral titer decay for a TIV model. Oseltamivir treatment is applied at various times post infection and at different efficacies. Top graph shows the effect of different treatments on the base viral infection parameters. The effect of varying the base infection parameters is also explored by either decreasing (bottom row) or increasing (upper row) viral production rate (p) (first column), infection rate (β) (second column), viral clearance rate (c) (third column), infectious lifespan (τ_I) (last column) by 10-fold compared to the base parameters.

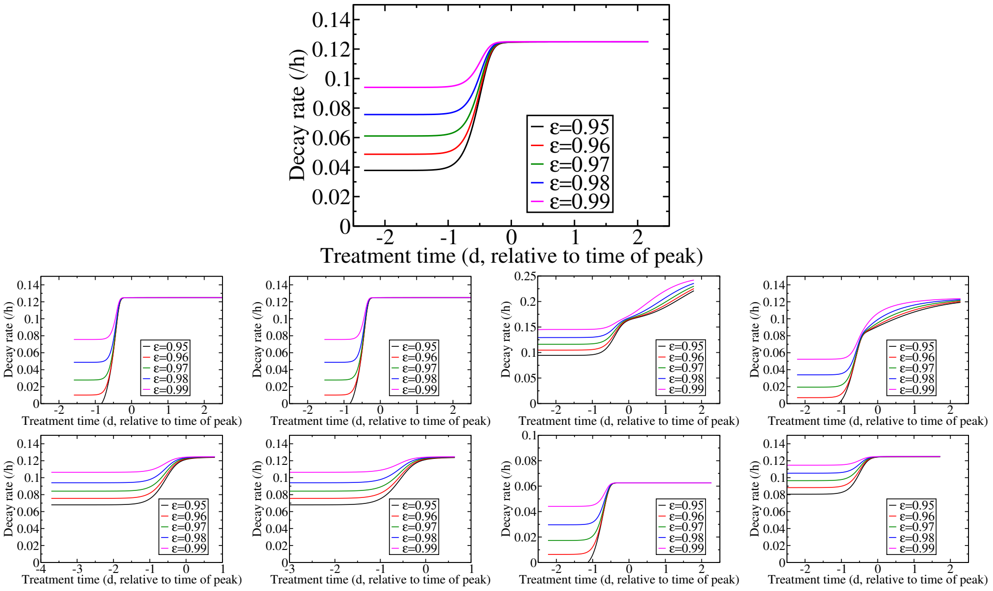

Figure 4: Effect of treatment delay on viral titer decay for a TIV model. Oseltamivir treatment is applied at various times post infection and at different efficacies. The effect of varying the base infection parameters is also explored by either decreasing (bottom row) or increasing (top row) viral production rate (p) (first column), infection rate (β) (second column), viral clearance rate (c) (third column), eclipse duration (τ_E) (fourth column), infectious lifespan (τ_I) (last column) by 10-fold (except τ_E, see text) compared to the base parameters.

References

1. **Manicassamy, B., S. Manicassamy, A. Belicha-Villanueva, G. Pisanelli, B. Pulendran, and A. Garca-Sastre**. 2010. Analysis of in vivo dynamics of influenza virus infection in mice using a GFP reporter virus. Proc. Natl. Acad. Sci. U.S.A. **107**:11531–11536.

2. **Tan, Z., S. Akerstrom, B. Y. Wee, S. K. Lal, A. Mirazimi, and Y.-J. Tan**. 2010. A new panel of NS1 antibodies for easy detection and titration of influenza A virus. J. Med. Virol. **82**:467–475.

3. **Rahim, M. N., M. Selman, P. J. Sauder, N. E. Forbes, W. Stecho, W. Xu, M. Lebar, E. G. Brown, and K. M. Coombs**. 2013. Generation and characterization of a new panel of broadly reactive anti-ns1 mabs for detection of influenza A virus. J. Gen. Virol. **94**:593–605.

4. **Baccam, P., C. Beauchemin, C. A. Macken, F. G. Hayden, and A. S. Perelson**. 2006. Kinetics of influenza A virus infection in humans. J. Virol. **80**:7590–7599.
